# The development of mouthwashes without anti-gonococcal activity for controlled clinical trials: an in vitro study

**DOI:** 10.12688/f1000research.20399.2

**Published:** 2020-02-17

**Authors:** Christophe Van Dijck, Vicky Cuylaerts, Piet Sollie, Anna Spychala, Irith De Baetselier, Jolein Laumen, Tania Crucitti, Chris Kenyon

**Affiliations:** 1 Department of Clinical Sciences, Institute of Tropical Medicine, Antwerp, Antwerp, 2000, Belgium; 2 Pharmacy Sollie, Antwerp, 2000, Belgium; 3 University of Cape Town, Cape Town, South Africa

**Keywords:** Neisseria gonorrhoeae, gonorrhea, pharyngitis, gargle, treatment, eradication, sexually transmitted diseases, placebo, randomized clinical trial, mouthwash

## Abstract

**Background**: The oropharynx plays a major role in the development and spread of antimicrobial resistant *Neisseria gonorrhoeae* among men who have sex with men. Trials are currently assessing the efficacy of bactericidal mouthwashes as possible therapeutic or preventive options against these pharyngeal gonococcal infections. Controlled clinical trials require the use of a placebo mouthwash without anti-gonococcal activity. So far, no such placebo mouthwash has been described. We describe the development of a mouthwash for this purpose.

**Methods**: The *in vitro *anti-gonococcal activity of Corsodyl®, Listerine Cool Mint®, Biotene®, phosphate buffered saline and six in-house placebo mouthwashes was evaluated. Three gonococcal isolates from patients with pharyngeal infection were exposed to the mouthwashes for a duration ranging from 30 seconds to 60 minutes. Isolates were then plated in duplicate onto blood agar (5% horse blood) and incubated for 24 hours (5-7% CO _2_, 35 ± 2°C). Growth of *N. gonorrhoeae* was scored on a five-point scale (0 = no growth, to 4 = confluent growth of colonies).

**Results**: Corsodyl® and Listerine Cool Mint® were bactericidal to all isolates. For the other mouthwashes, the median growth score after 60 minutes of exposure was 4 (interquartile range 4-4) for phosphate buffered saline; 1 (interquartile range 1-3) for Biotene®; and ranged between 0 and 2 for the in-house composed mouthwashes. An in-house composed mouthwash (Placebo 6) performed best, with a growth score of 2.5 (interquartile range 1-3).

**Conclusions**: All the evaluated potential placebo mouthwashes were bacteriostatic after gonococcal exposure of 30 to 60 minutes. In-house composed Placebo 6 showed less inhibition on gonococcal growth than Biotene® and the other in-house placebos and demonstrates, in our opinion, a good trade-off between anti-gonococcal properties and taste.

## Introduction

The importance of antimicrobial resistance (AMR) in *Neisseria gonorrhoeae* cannot be overstated. The bacterium is renowned for its capability to acquire AMR and has developed resistance to all classes of antimicrobials used for its treatment ^1^. AMR frequently emerges in core groups, such as men who have sex with men (MSM) ^2^. The pharmaco-ecological theory of AMR states that this resistance is driven by two main factors: (a) frequent transmission of gonococci between individuals within a densely interconnected sexual network, and (b) excessive antimicrobial use which acts as a selection pressure on circulating gonococci to acquire AMR ^3– 5^. If this theory is correct, current efforts to reduce sexually transmitted infection (STI) prevalence via expanded screening and antimicrobial therapy in MSM may paradoxically be playing an important role in the promotion of gonococcal AMR ^5, 6^.

These considerations have led to efforts to reduce the prevalence of gonococci in MSM and other core groups with non-antimicrobial products. One option is the use of an antiseptic mouthwash to decrease the oropharyngeal prevalence of gonococci (and other STIs). A modeling study showed that regular use of a mouthwash by MSM could reduce the prevalence of gonococci at different body sites ^7^. A further consideration is that the oropharynx plays a central role in the emergence and spread of gonococcal AMR among MSM because of multiple reasons, which are reviewed elsewhere ^8^. If a mouthwash can reduce the prevalence of oropharyngeal gonorrhoea without selecting for AMR, this may have the added benefit of reducing the probability of AMR emerging at this site ^4^. Two randomized controlled trials (RCTs) are currently underway to assess whether regular mouth washing and gargling in MSM is able to reduce the cumulative incidence of gonorrhoea and other STIs. The OMEGA (Oral Mouthwash use to Eradicate GonorrhoeA) study is an RCT that assesses whether daily use of Listerine Zero ^®^ can reduce the incidence of pharyngeal gonorrhoea in a population of Australian MSM ( ACTRN12616000247471) ^9^. We are conducting a second RCT to assess if the use of Listerine Cool Mint ^®^ (LCM) is able to reduce the cumulative incidence of gonorrhoea (PReGo – Preventing Resistance in Gonorrhoea Study; registered at ClinicalTrials.gov with the identifier NCT03881007).

The choice of an optimal placebo is critical to the success of these RCTs. It is particularly important that a placebo is inert and has no bactericidal or bacteriostatic effect on gonococci. If it did, it would increase the probability of a false negative study outcome.

So far, no study has assessed placebo mouthwashes for this purpose. In this paper, we describe the process of developing and testing a series of candidate placebo mouthwashes. Our aim was to find the most suitable formulation for use as a placebo in the PReGo study. The major criterion we used to assess the mouthwash was its anti-gonococcal activity.

## Methods

### Isolates

We used three stored isolates of *Neisseria gonorrhoeae* that had been previously isolated from the oropharynx of three treatment-naive women with pharyngeal infection at the STI clinic of the Institute of Tropical Medicine, Antwerp, as part of routine gonococcal surveillance monitoring. The isolates were preserved in skimmed milk and 20% glycerol at -80°C until the experiments were performed. Antimicrobial susceptibility was determined by the agar dilution method according to Clinical & Laboratory Standards Institute ^10^.

### Mouthwashes

The commercially available products Listerine Cool Mint ^®^ (LCM, containing alcohol and essential oils) and Corsodyl ^®^ (containing chlorhexidine 0.2%) were used to assess the isolate’s susceptibility to antibacterial mouthwashes.

Biotene ^®^, a commercially available mouthwash that does not contain alcohol, essential oils or chlorhexidine, was expected to have no antibacterial effect and was thus the first mouthwash to be evaluated as a potential placebo substance.

Subsequently, six other potential placebo mouthwashes were manufactured by a pharmacist (Sollie Pharmacy, Antwerp) based on readily available and inexpensive ingredients that are stable at room temperature. Ingredients added to create a medicinal taste were sorbitol, sodium saccharinate, benzoic acid, ethanol, mint spiritus, raspberry extract and/or elderberry extract; ingredients added as a colorant were malachite green, raspberry extract, elderberry extract or solutio viridis. Only mouthwashes with a medicinal taste, as appreciated by one of the researchers (CK), were included in the experiment. The composition of the mouthwashes is displayed in Table 1 and Table 2. Based on the properties of these ingredients, no major side effects would be expected to occur.

**Table 1.** Ingredients of the commercially available mouthwashes, according to their product insert.

Mouthwash	Ingredients
**Biotene ^®^**	purified water, glycerin, xylitol, sorbitol, propylene glycol, poloxamer 407, sodium benzoate, hydroxyethyl cellulose, methylparaben, propylparaben, flavor, sodium phosphate and disodium phosphate
**Listerine Cool** **Mint ^®^**	aqua, alcohol 21.6%, sorbitol, poloxamer 407, benzoic acid, sodium saccharin, eucalyptol 0.092%, aroma, methyl salicylate 0.06%, thymol 0.064%, menthol 0.042%, sodium benzoate, flavor, green 3
**Corsodyl ^®^**	chlorhexidine digluconate 0.2%, ethanol, peppermint flavour, polyoxyl hydrogenated castor oil, sorbitol, cochenille red dye (E 124), purified water

**Table 2.** Ingredients of the in-house mouthwashes.

Mouthwash	Ingredients										
	Sorbitol (g)	Sodium saccharinate (g)	Benzoic acid (g)	Ethanol 96% (g)	Mint spiritus (g)	Malachite green ^§^ (g)	Raspberry extract (g)	Elderberry extract (g)	Solutio viridis ^$^ (g)	Aqua conservans * (g)	Total (g)
**Placebo 1**	30.00	0.10	0.20	10.00	1.10	1.75				156.85	200
**Placebo 2**	30.00	0.10			0.66	1.75				167.49	200
**Placebo 3**	30.00	0.10				1.75	1.00			167.15	200
**Placebo 4**	30.00	0.05					1.00			168.95	200
**Placebo 5**	30.00	0.05						2.00		167.95	200
**Placebo 6**	30.00	0.10							0.70	169.20	200

^§^ 100 g Malachite green contains: 0.01 g malachite green oxalate, 99.99 g aqua conservans. ^$^ 100 g Solutio viridis contains: 0.3 g patent blue (E131), 0.3 g tartrazine (E102), 0.15 g sodium benzoic acid, 0.1 g tartaric acid, 99.15 g purified water. * 100 g Aqua conservans contains: 0.0724 g methylparahydroxybenzoate, 0.0310 g propylparahydroxybenzoate, 0.9959 g propylene glycol, 98.901 g purified water.

Phosphate buffered saline (PBS, pH 7.3 ± 0.2) was used as a negative control (inert product maintaining gonococcal viability) during every experiment.

### Assessment of antibacterial effect

**Figure 1.**
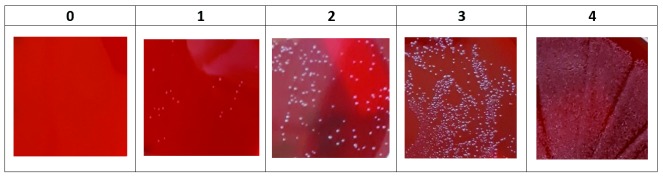
Five-point scale for scoring Neisseria gonorrhoeae growth on blood agar (0 = no growth; 1 = some colonies; 2 = numerous colonies; 3 = entire agar plate covered with colonies; 4 = confluent growth of colonies).

Each gonococcal isolate was brought into suspension in 3mL PBS at a 0.5 to 0.8 McFarland turbidity, corresponding to a concentration of 10 ^8^ CFU/mL. From these suspensions, 100μl was then added to 900µL of each mouthwash, resulting in a concentration of 10 ^7^ CFU/mL. After 30 seconds, 60 seconds, five minutes, 30 minutes and 60 minutes at ambient temperature (20 ± 5°C), 10µL aliquots were plated onto blood agar (5% horse blood) and incubated for 24 hours in a 6 ± 1% CO _2_ environment at 35 ± 2°C. Bacterial growth was visually scored on a semi-quantitative five-point scale, as described in Figure 1. Plating was conducted in duplicate for each isolate and all bacterial growth assessments were made by a single observer.

No statistical analysis was performed. This study did not involve any experiments on humans or animals and thus no ethical clearance was required.

## Results

All three isolates were susceptible to ceftriaxone and spectinomycin; isolates B and C had a slightly increased minimum inhibitory concentration (MIC) for azithromycin and isolate A had a high MIC for ciprofloxacin and cefixime. None of the strains produced penicillinase ( Table 3).

**Table 3.** Antimicrobial susceptibility of *Neisseria gonorrhoeae* isolates used in the experiment.

Isolate	MIC values (mg/L)					
	Ciprofloxacin	Ceftriaxone	Azithromycin	Spectinomycin	Cefixime	Penicillinase
**A**	16.000	0.030	0.250	16.000	0.250	negative
**B**	0.004	0.008	0.500	16.000	0.015	negative
**C**	0.004	0.008	0.500	16.000	0.015	negative

MIC, Minimum Inhibitory Concentration; determined by agar dilution method according to Clinical & Laboratory Standards Institute.

All isolates were fully susceptible to LCM and Corsodyl ^®^; a full bactericidal effect was observed after an exposure of 30 seconds or longer ( Table 4) ^11^.

**Table 4.** Growth of *Neisseria gonorrhoeae* after exposure to the mouthwashes.

Mouthwash	N	Median growth score (IQR) after exposure during				
		30 seconds	60 seconds	5 minutes	30 minutes	60 minutes
Listerine Cool Mint ^®^	6	0 (0-0)	0 (0-0)	NA	NA	NA
Corsodyl ^®^	6	0 (0-0)	0 (0-0)	NA	NA	NA
Biotene ^®^	6	4 (4-4)	4 (4-4)	4 (2-4)	1 (1-3)	1 (1-3)
Placebo 1	6	1 (0-2)	1 (0-1)	NA	NA	NA
Placebo 2	6	4 (4-4)	4 (4-4)	3 (3-4)	3 (1-3)	1 (0-2)
Placebo 3	6	2 (2-2)	0 (0-0)	0 (0-0)	0 (0-0)	0 (0-0)
Placebo 4	6	2 (1-2)	0 (0-0)	0 (0-0)	0 (0-0)	0 (0-0)
Placebo 5	6	4 (4-4)	4 (3-4)	3 (2-3)	0 (0-0)	0 (0-0)
Placebo 6	6	4 (4-4)	4 (4-4)	4 (3-4)	3 (2-4)	2.5 (1-3)
PBS	6	4 (4-4)	4 (4-4)	4 (4-4)	4 (4-4)	4 (4-4)

NA, not assessed; IQR, interquartile range; PBS, phosphate buffered saline.

Exposure to Biotene ^®^ for 30 minutes or longer was found to inhibit gonococcal growth considerably ( Table 4).

Placebo 1, an ethanol-containing mouthwash was designed to have a similar color and taste as LCM ^®^ but led to almost complete inhibition of gonococcal growth even after a short duration of exposure. Placebo 2 contained no ethanol and a lower amount of mint spiritus. Yet, its bacteriostatic effect was comparable to Biotene ^®^. In order to determine if mint spiritus or malachite green were the inhibiting factors, these ingredients were sequentially omitted in Placebo 3 and 4. Raspberry extract was added to both in order to improve the taste, but this resulted in strong inhibition of gonococcal growth in both cases. Placebo 5 contained elderberry extract instead, but substantial gonococcal growth inhibition was seen here, too. Placebo 6 contained another type of colorant (solutio viridis) and showed the least bacteriostatic effect after 30 and 60 minutes of exposure ( Table 4). During every experiment, there was full and confluent gonococcal growth after exposure to the negative control substance (PBS) ( Table 4).

We noted a slight difference in susceptibility to the mouthwashes between the three tested gonococcal isolates. Isolate A was more susceptible to placebos 1–6 and Biotene ^®^ compared with isolates B and C. However, all strains showed equivalent susceptibility to LCM and Corsodyl ^®^ ( Table 5 and Table 6). These differences were not assessed for statistical significance.

**Table 5.** Growth of *Neisseria gonorrhoeae* after exposure to seven potential placebo mouthwashes (Biotene ^®^ and Placebo 1-6).

Isolate	N	Median growth score (IQR) after exposure during				
		30 seconds	60 seconds	5 minutes	30 minutes	60 minutes
**A**	14	3.5 (2-4)	3 (0-4)	1 (0-3)	0.5 (0-1)	0 (0-1)
**B**	14	4 (2-4)	4 (0-4)	2.5 (0-4)	0.5 (0-2.5)	0.5 (0-1.5)
**C**	14	4 (1-4)	4 (0-4)	3.5 (0-4)	1.5 (0-3)	0.5 (0-3)

IQR, interquartile range.

**Table 6.** Growth of *Neisseria gonorrhoeae* after exposure to the mouthwashes.

Isolate	Mouthwash	N	Median growth score (IQR) after exposure during				
			30 seconds	60 seconds	5 minutes	30 minutes	60 minutes
**A**	Listerine Cool Mint ^®^	2	0 (0-0)	0 (0-0)	NA	NA	NA
	Corsodyl ^®^	2	0 (0-0)	0 (0-0)	NA	NA	NA
	Biotene ^®^	2	4 (4-4)	4 (4-4)	3 (3-3)	1 (1-1)	1 (1-1)
	Placebo 1	2	2 (2-2)	1 (1-1)	NA	NA	NA
	Placebo 2	2	4 (4-4)	4 (4-4)	3 (3-3)	1 (1-1)	0 (0-0)
	Placebo 3	2	2 (2-2)	0 (0-0)	0 (0-0)	0 (0-0)	0 (0-0)
	Placebo 4	2	2 (2-2)	0 (0-0)	0 (0-0)	0 (0-0)	0 (0-0)
	Placebo 5	2	4 (4-4)	3 (3-3)	2 (2-2)	0 (0-0)	0 (0-0)
	Placebo 6	2	4 (4-4)	4 (4-4)	3 (3-3)	3 (3-3)	2 (2-2)
	PBS	2	4 (4-4)	4 (4-4)	4 (4-4)	4 (4-4)	4 (4-4)
**B**	Listerine Cool Mint ^®^	2	0 (0-0)	0 (0-0)	NA	NA	NA
	Corsodyl ^®^	2	0.5 (0-1)	0 (0-0)	NA	NA	NA
	Biotene ^®^	2	4 (4-4)	4 (4-4)	2 (2-2)	1 (1-1)	1 (1-1)
	Placebo 1	2	0 (0-0)	0 (0-0)	NA	NA	NA
	Placebo 2	2	4 (4-4)	4 (4-4)	4 (4-4)	3 (3-3)	2 (2-2)
	Placebo 3	2	2 (2-2)	0 (0-0)	0 (0-0)	0 (0-0)	0 (0-0)
	Placebo 4	2	2 (2-2)	0 (0-0)	0 (0-0)	0 (0-0)	0 (0-0)
	Placebo 5	2	4 (4-4)	4 (4-4)	3 (3-3)	0 (0-0)	0 (0-0)
	Placebo 6	2	4 (4-4)	4 (4-4)	4 (2-4)	2 (2-2)	1 (1-1)
	PBS	2	4 (4-4)	4 (4-4)	4 (4-4)	4 (4-4)	4 (4-4)
**C**	Listerine Cool Mint ^®^	2	0 (0-0)	0 (0-0)	NA	NA	NA
	Corsodyl ^®^	2	0 (0-0)	0 (0-0)	NA	NA	NA
	Biotene ^®^	2	4 (4-4)	4 (4-4)	4 (4-4)	3 (3-3)	3 (3-3)
	Placebo 1	2	1 (1-1)	1 (1-1)	NA	NA	NA
	Placebo 2	2	4 (4-4)	4 (4-4)	4 (4-4)	3 (3-3)	1 (1-1)
	Placebo 3	2	1 (1-1)	0 (0-0)	0 (0-0)	0 (0-0)	0 (0-0)
	Placebo 4	2	2 (2-2)	0 (0-0)	0 (0-0)	0 (0-0)	0 (0-0)
	Placebo 5	2	4 (4-4)	4 (4-4)	3 (3-3)	0 (0-0)	0 (0-0)
	Placebo 6	2	4 (4-4)	4 (4-4)	4 (4-4)	4 (4-4)	3 (3-3)
	PBS	2	4 (4-4)	4 (4-4)	4 (4-4)	4 (4-4)	4 (4-4)

NA, not assessed; PBS, phosphate buffered saline.

## Discussion

The recognition of the oropharynx as a source of gonococcal transmission and the genesis of antimicrobial resistance in groups such as MSM has directed research interest towards novel non-antimicrobial methods to prevent or treat oropharyngeal gonococcal infection. Mouthwashes are one such option. In order to determine the efficacy of an intervention involving the use of a mouthwash, RCTs should be performed, and a non-bactericidal placebo is a prerequisite for these trials.

Commercially available non-alcohol containing mouthwashes (like Biotene ^®^) are an attractive option, but our experiments suggest that exposure to Biotene ^®^ for longer than five minutes may inhibit the growth of gonococci. Mouthwashes are typically used for 60 seconds but the substantivity of its ingredients may result in the antibacterial activity of mouthwashes persisting for over six hours ^12, 13^. To optimize their STI preventive potential, mouthwashes could be used pre and post sex, which could lead to multiple exposures per day. These considerations triggered the search for a placebo with minimal inhibitory effect for periods of up to 60 minutes.

All three isolates in the experiment were fully susceptible to LCM and Corsodyl ^®^ and isolate A was most susceptible to all placebo mouthwashes. Although this difference in susceptibility may have been the result of random variability, we could speculate that, in the absence of overt resistance to antiseptics, there might be a mechanism that partially protected isolate B and C from the harmful effect of some of the mouthwash constituents. Isolates B and C had a reduced susceptibility to azithromycin. This might have been due to the increased expression of an efflux mechanism such as the Mtr (multiple transferable resistance) efflux pump, which is linked to resistance to macrolides, as well as to many other substances like dyes and detergents ^14^. We did, however, not perform genotypic assessment of the isolates used in this experiment.

After testing multiple combinations of ingredients, we found that Placebo 6 had the least bacteriostatic effect *in vitro.*

The limitations of this study include the following. First, budget and time constraints did not allow to perform any *in vivo* evaluation of the mouthwashes. It is of particular interest to know whether participants can distinguish Placebo 6 from an antibacterial mouthwash. A formal head-to-head comparison with LCM is planned as part of the PReGo study. Second, the sample size of the current experiment was too small to statistically assess differences between isolates and between mouthwashes. We may have over- or underestimated the true effect of the mouthwashes. Additionally, the experiments were performed sequentially, which may have introduced some inter-run variation. In each experiment we did however include a PBS exposed control. and plating was done in duplicate. Third, the observer who assessed bacterial growth was not blinded to the ingredients of the mouthwashes, we did not use a validated quantitative assessment method and we did no further *in vivo* or *in vitro* fitness testing of the isolates after exposure to the mouthwashes. Fourth, we used isolates from women with pharyngeal gonococcal infection, which are possibly not representative of the gonococci circulating among MSM. Their susceptibility pattern was, however, similar to that observed in most gonococcal isolates from MSM. Fifth, our *in vitro* findings are not necessarily representative of the *in vivo* setting as anatomical and biological properties may influence the effect of a mouthwash against gonococci in the throat. Bioactive molecules in saliva may, for example, have synergistic or antagonistic effects on the mouthwash’s active ingredients. Finally, we did not assess the effect of the placebo on the oropharyngeal microbiome. An increased or decreased growth of other oropharyngeal commensals might theoretically compete with gonococcal proliferation in the throat and influence gonococcal infectivity as well. 

## Conclusion

This experiment has shown that it is hard to develop an ideal placebo mouthwash as a range of frequently used ingredients inhibit gonococcal growth. A commercially available mouthwash like Biotene ^®^ seemed the perfect option at first but it had a bacteriostatic effect. A process of serial testing of various placebos resulted in a placebo mouthwash, which we believe demonstrates a good trade-off between anti-gonococcal properties and taste.

## Data availability

### Underlying data

Figshare: *In vitro* gonococcal growth after exposure to mouthwashes. https://doi.org/10.6084/m9.figshare.9757859 ^11^.

This project contains the following underlying data:

- Data.xlsx (spreadsheet containing raw growth scores of the individual isolates after exposure to the experimental substances)

Data are available under the terms of the Creative Commons Zero "No rights reserved" data waiver (CC0 1.0 Public domain dedication).

## References

[1] Unemo M Del Rio C Shafer WM: Antimicrobial Resistance Expressed by *Neisseria gonorrhoeae*: A Major Global Public Health Problem in the 21 ^st^ Century. *Microbiol Spectr.* 2016;4(3):213–37. 10.1128/microbiolspec.EI10-0009-2015 27337478 PMC4920088

[2] Lewis DA: The role of core groups in the emergence and dissemination of antimicrobial-resistant *N gonorrhoeae*. *Sex Transm Infect.* 2013;89 Suppl 4:iv47–51. 10.1136/sextrans-2013-051020 24243880

[3] Kenyon C Osbak K: Certain attributes of the sexual ecosystem of high-risk MSM have resulted in an altered microbiome with an enhanced propensity to generate and transmit antibiotic resistance. *Med Hypotheses.* 2014;83(2):196–202. 10.1016/j.mehy.2014.04.030 24857261

[4] Kenyon CR Schwartz IS: Effects of Sexual Network Connectivity and Antimicrobial Drug Use on Antimicrobial Resistance in *Neisseria gonorrhoeae*. *Emerg Infect Dis.* 2018;24(7):1195–1203. 10.3201/eid2407.172104 29912682 PMC6038757

[5] Kenyon C: Risks of Antimicrobial Resistance in *N. gonorrhoeae* Associated with Intensive Screening Programs in Pre-Exposure Prophylaxis Programs. *Clin Infect Dis.* 2018;67(1):154–155. 10.1093/cid/ciy048 29370373

[6] Kenyon C: We need to consider collateral damage to resistomes when we decide how frequently to screen for chlamydia/gonorrhoea in preexposure prophylaxis cohorts. *AIDS.* 2019;33(1):155–157. 10.1097/QAD.0000000000002020 30234609

[7] Zhang L Regan DG Chow EPF: *Neisseria gonorrhoeae* Transmission Among Men Who Have Sex With Men: An Anatomical Site-Specific Mathematical Model Evaluating the Potential Preventive Impact of Mouthwash. *Sex Transm Dis.* 2017;44(10):586–592. 10.1097/OLQ.0000000000000661 28876289

[8] Lewis DA: Will targeting oropharyngeal gonorrhoea delay the further emergence of drug-resistant *Neisseria gonorrhoeae* strains? *Sex Transm Infect.* 2015;91(4):234–7. 10.1136/sextrans-2014-051731 25911525

[9] Chow EPF Walker S Hocking JS: A multicentre double-blind randomised controlled trial evaluating the efficacy of daily use of antibacterial mouthwash against oropharyngeal gonorrhoea among men who have sex with men: the OMEGA (Oral Mouthwash use to Eradicate GonorrhoeA) study protocol. *BMC Infect Dis.* 2017;17(1):456. 10.1186/s12879-017-2541-3 28659133 PMC5490220

[10] CLSI: Methods for Dilution Antimicrobial Susceptibility Tests for Bacteria That Grow Aerobically. 11th ed. CLSI standard M07. Wayne, PA: Clinical and Laboratory Standards Institute.2018 Reference Source

[11] Van Dijck C: *In vitro* gonococcal growth after exposure to mouthwashes.2019 10.6084/m9.figshare.9757859.v1

[12] Prada-Lopez I Quintas V Donos N: *In situ* substantivity of the essential oils in the oral cavity. *Microb Pathog Strateg Combat them Sci Technol Educ*(A Méndez-Vilas, Ed).2013;1112–1122. Reference Source

[13] Tomás I Cousido MC García-Caballero L: Substantivity of a single chlorhexidine mouthwash on salivary flora: influence of intrinsic and extrinsic factors. *J Dent.* 2010;38(7):541–6. 10.1016/j.jdent.2010.03.012 20380865

[14] Morse SA Lysko PG McFarland L: Gonococcal strains from homosexual men have outer membranes with reduced permeability to hydrophobic molecules. *Infect Immun.* 1982;37(2):432–8. 6811431 10.1128/iai.37.2.432-438.1982 PMC347552

