# Supplementary material for: The development of mouthwashes without anti-gonococcal activity for controlled clinical trials: an in vitro study
Source: F1000Res. 2020 Feb 17;8:1620. Originally published 2019 Sep 11. [Version 2] doi: 10.12688/f1000research.20399.2 (PMC7001748; doi:10.12688/f1000research.20399.2)
Supplement: Peer review history

# Peer review: Reviewer response for version 1

**DOI:** 10.5256/f1000research.22418.r58822

**Published:** 2020-02-04

**Authors:** Victoria Miari

**Affiliations:** 1 Department of Infectious and Tropical Diseases, London School of Hygiene & Tropical Medicine, London, UK

**Overall**

- This study is worthwhile as I'm aware it is difficult to design trials with adequate control solutions. 
- The study was written well and clearly for the most part; I have included some comments for improvement.

**Abstract**

- Please make clear that you are developing a "control" mouthwash rather than an active one.
- When you say the experiments were performed in duplicate you suggest that the experiment was performed twice. Reading on, you mention that you performed two measurements from the same experiment which is not an experimental duplicate. Can you rephrase to reflect this? You also mentioned that there is a good trade-off between anti-gonococcal activity and taste for one placebo; how did you measure this? It is not described in the main study.

**Methods**

- You mention PBS allows for unrestricted gonococcal growth; I do not believe this is correct; it maintains viability but not growth. 
- Initial colony counts were not performed; this would have allowed comparison between the different mouthwashes with higher confidence. You cold have also somehow measured reduction in viability; this will need to be justified in the discussion. 
- You present results for penicillinase; how did you test this.

**Results**

- Can you please put title rows on table 1? 
- For Table 2 it would be helpful to mention what the final volume/weight/mass the placebos were, or enter the values as percentages? 
- Table 6 could perhaps go in supplementary files? The summary tables enough for these results.

**Discussion**

- What do you mean when you say you did not assess bacterial growth "blindly"? See comments in methods for what else needs to be considered in discussion.
- You also mentioned experiments performed sequentially and also plating was performed in duplicate; from the results it seems like one of these was done. Can you clarify this?

I confirm that I have read this submission and believe that I have an appropriate level of expertise to confirm that it is of an acceptable scientific standard.

# Peer review: Reviewer response for version 1

**DOI:** 10.5256/f1000research.22418.r55772

**Published:** 2019-11-11

**Authors:** Maarten Franciscus Schim van der Loeff

**Affiliations:** 1 Department of Infectious Diseases, Public Health Service Amsterdam, Amsterdam, The Netherlands

This report by Van Dijck et al. seeks to evaluate the anti-gonococcal activity of several commercially available mouth washes, as well as of one commercial and several in-house produced 'placebo' mouth washes. The authors find that all tested commercially available mouth washes have some anti-gonococcal activity, and that some of the 'placebo' mouth washes did as well. 

 This study is important as mouth washes have been suggested as a potential tool for prevention of gonorrhoea on an individual and a population level. Trials examining the efficacy of oral mouth wash need a 'placebo' without anti-gonococcal activity. This report provides important data towards that.

 This brief report is clearly written. The conclusions are based on the data. The limitations of the small study are clearly described in the Discussion.

 I am a physician and epidemiologist and recommend that also a microbiologist should review the manuscript.

 I have a few minor comments:

1. In the abstract it is not clear how the 5-point scale of *N. gonorrhoeae* growth is to be interpreted; make it explicit that 0 means no growth and 4 extensive growth.
2. The abstract mentions an IQR of 2-3 for placebo 6 at 60 minutes; Table 4 mentions an IQR of 1-3. Please check and correct.
3. Not all readers may be familiar with the term "pharmaecological" (perhaps better spelled as "pharma-ecological"?) so a fuller explanation may be helpful.

I confirm that I have read this submission and believe that I have an appropriate level of expertise to confirm that it is of an acceptable scientific standard.

# Response

**Published:** 2020-02-12

**Authors:** Christophe Van Dijck

**Affiliations:** Institute of Tropical Medicine, Antwerp, Belgium

We are very thankful to the reviewer for his valuable comments.

**Comment 1. In the abstract it is not clear how the 5-point scale of *N. gonorrhoeae* growth is to be interpreted; make it explicit that 0 means no growth and 4 extensive growth.**

Response: The abstract has been changed accordingly

**Comment 2. The abstract mentions an IQR of 2-3 for placebo 6 at 60 minutes; Table 4 mentions an IQR of 1-3. Please check and correct.**

Response: Thank you for pointing out this error. It has been corrected.

**Comment 3. Not all readers may be familiar with the term "pharmaecological" (perhaps better spelled as "pharma-ecological"?) so a fuller explanation may be helpful.**

Response: The term pharmaecological has been changed into “pharmaco-ecological” and the sentence has been rephrased to clarify the term.
